# Physical analysis of an Antarctic ice core—towards an integration of micro- and macrodynamics of polar ice*

**DOI:** 10.1098/rsta.2015.0347

**Published:** 2017-02-13

**Authors:** Ilka Weikusat, Daniela Jansen, Tobias Binder, Jan Eichler, Sérgio H. Faria, Frank Wilhelms, Sepp Kipfstuhl, Simon Sheldon, Heinrich Miller, Dorthe Dahl-Jensen, Thomas Kleiner

**Affiliations:** 1 AWI-Glaciology, Alfred-Wegener-Institute Helmholtz-Centre for Polar and Marine Research, Bremerhaven, Germany; 2 Department of Geosciences, Eberhard Karls University, Tübingen, Germany; 3 BC3-Basque Centre for Climate Change, Ikerbasque, Bilbao, Spain; 4 NUT-Nagaoka University of Technology Nagaoka, Niigata, Japan; 5 Georg-August-Universität Göttingen, Göttingen, Germany; 6 CIC, Niels Bohr Institute, University of Copenhagen, Copenhagen, Denmark

**Keywords:** polar ice core, microstructure, borehole deformation, fabric, texture, ice flow modelling

## Abstract

Microstructures from deep ice cores reflect the dynamic conditions of the drill location as well as the thermodynamic history of the drill site and catchment area in great detail. Ice core parameters (crystal lattice-preferred orientation (LPO), grain size, grain shape), mesostructures (visual stratigraphy) as well as borehole deformation were measured in a deep ice core drilled at Kohnen Station, Dronning Maud Land (DML), Antarctica. These observations are used to characterize the local dynamic setting and its rheological as well as microstructural effects at the EDML ice core drilling site (European Project for Ice Coring in Antarctica in DML). The results suggest a division of the core into five distinct sections, interpreted as the effects of changing deformation boundary conditions from triaxial deformation with horizontal extension to bedrock-parallel shear. Region 1 (uppermost approx. 450 m depth) with still small macroscopic strain is dominated by compression of bubbles and strong strain and recrystallization localization. Region 2 (approx. 450–1700 m depth) shows a girdle-type LPO with the girdle plane being perpendicular to grain elongations, which indicates triaxial deformation with dominating horizontal extension. In this region (approx. 1000 m depth), the first subtle traces of shear deformation are observed in the shape-preferred orientation (SPO) by inclination of the grain elongation. Region 3 (approx. 1700–2030 m depth) represents a transitional regime between triaxial deformation and dominance of shear, which becomes apparent in the progression of the girdle to a single maximum LPO and increasing obliqueness of grain elongations. The fully developed single maximum LPO in region 4 (approx. 2030–2385 m depth) is an indicator of shear dominance. Region 5 (below approx. 2385 m depth) is marked by signs of strong shear, such as strong SPO values of grain elongation and strong kink folding of visual layers. The details of structural observations are compared with results from a numerical ice sheet model (PISM, isotropic) for comparison of strain rate trends predicted from the large-scale geometry of the ice sheet and borehole logging data. This comparison confirms the segmentation into these depth regions and in turn provides a wider view of the ice sheet.

This article is part of the themed issue ‘Microdynamics of ice’.

## 1. Introduction

Variations in net mass transport of ice towards the ocean lead to variations in ice flux and eventually sea-level variations. Ice sheets possess the highest potential to cause drastic changes within the global water cycle owing to their large water reservoir. This horizontal movement of ice is expressed in the surface velocities measured locally with ground-based GPS surveys [1] or by remote sensing techniques on larger scales [2]. The observed surface velocities, however, result from a superposition of several components: (i) basal sliding owing to a temperate base of the ice, (ii) possible deformation of the bed material (sediments, glacial till), and (iii) the internal deformation of the material ice itself. While the former two components play an important role at the margins of the polar ice sheets, especially in fast-flowing outlet glaciers or ice streams, the internal deformation of ice is the main component in the interior of Antarctica and Greenland. In the upper part of the ice column, vertical thinning is the dominating process. The major ice deformation component in the geographically horizontal direction, that is, the direction towards the ocean, is shearing on horizontal planes owing to friction at the interface between ice and bedrock. This horizontal shearing becomes the dominant deformation component with depth. The balance between the different components of ice deformation contributing to horizontal transport is difficult to determine as the available data originate mainly from surface observations. As a first approximation, the Dansgard–Johnsen [3] assumption is often considered, which assumes a constant vertical strain rate in the upper two-thirds of the ice column, which then decreases linearly to no vertical deformation at the frozen bed. Thus, in general, in the lower third, the shear deformation is more or less the dominant process. This holds for the most typical and common locations in an ice sheet, therefore excluding extraordinary sites such as domes. Especially in areas with low ice flow velocities, this estimation appears to be a good approximation, reconfirmed by the simulated evolution of vertical strain rate profiles in up-to-date models. With respect to improved implementation of the crystalline lattice-preferred orientation (LPO) anisotropy effects in flow models [4–13], the exact identification and reconstruction of the relation between vertical compression and horizontal shear and its development with depth are required. An anisotropic description can lead to substantial feedback effects in terms of effective viscosity with respect to the principal deformation directions.

The contributions of vertical compression in the upper part and horizontal shear in the lower part of the ice sheet can be estimated, using information obtained from ice coring. Ice cores and their boreholes provide us with local insights into dynamical processes below the surface. The evolution of *c*-axis distributions (LPO) with depth is an indicator of the changing dominance of the deformation modes [14]. Thus, *c*-axis distributions measured from ice core samples directly represent the mechanical contribution to microdynamic processes. This information can be complemented by grain topology (size and shape) data also to represent the recrystallization (thermodynamic) contribution [15]. Grain size and grain shape are structural parameters that can be evaluated in order to support conclusions made from the LPO measurements with respect to deformation modes. Such characteristic grain size and shape microstructures are well known and also used as shear-sense indicators, e.g. as ‘oblique foliation’ in mylonitic rocks [16, p. 128]. Mylonites are rocks found in shear zones which experienced large ductile strain rate, but under colder homologous temperatures (*T*/*T*_m_) than ice. Owing to the high homologous temperature for natural ice in general (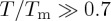) and the slow creep flow in most deep ice coring sites, such effects of deformation modes are very moderate and difficult to discern, because recrystallization strongly overprints deformation effects and masks typical deformation microstructures [17–20]. Furthermore, deformation mechanisms, such as small-scale folding initiated by grain kinking, provide new insights into the microdynamic processes with consequences for mesoscale [21] and possibly also macroscale structures [22]. The meso- to macroscale impact of changing deformation regimes with different deformation modes and mechanisms with depth can be monitored by repeatedly logging the borehole geometry after certain time intervals, which also reveals the onset of shear deformation dominance with depth. Comparison of the described observations with strain rate components predicted by a flow model can illuminate possible misconceptions in our understanding of large-scale ice flow.

The aim of this contribution is to revisit the idea of deformation modes at the EDML site from meso- and microstructural data [23] in combination with new observational data, which became available recently. We combine these with strain rate estimates provided by a large-scale three-dimensional flow model, and thus consider also a broader ambient flow field than the purely one-dimensional Dansgard–Johnsen [3] assumption. This combination provides us with a solid understanding of shear versus compressive deformation for the EDML ice core location.

### (a) Introduction—EDML ice core

**Figure 1.**
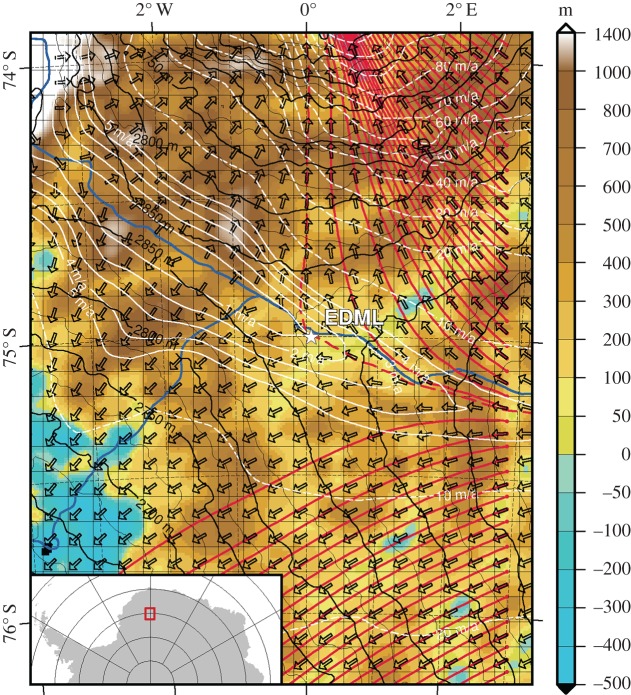
Map of the EDML ice divide area with input and output information of the ice flow model. The divide line is shown in blue [26]. White isolines and arrows are the magnitudes and directions of surface velocities, respectively, calculated by the model. The resulting streamlines seeded at the eastern margin of the domain are shown as solid red lines, whereas the streamline passing the grid location next to the EDML site (the location of the presented strain rates) is shown as the dashed red line. The blue to brown colour code represents the bedrock topography while black isolines show the surface elevation; both from the Bedmap2 dataset [27]. The square grid is the model grid with 10 km spacing.

The European Project for Ice Coring in Antarctica (EPICA) in the Dronning Maud Land (in short: EDML) ice core was drilled between 2001 and 2006 at the Kohnen Station, Antarctica (position 79°00′ S, 0°04′ E, elevation 2892 m.a.s.l.) [24,25]. The location is shown in figure 1 and was chosen because of the relatively high accumulation rate at the drill site, which enabled palaeoclimate proxy records to be obtained with a high temporal resolution. Furthermore, its location in the Atlantic sector of Antarctica allows for a comparison with the Greenland ice cores [28]. The core reached a length of 2774 m, penetrating down to just above the bedrock at 2782 m vertical ice thickness. The ice surface topography exhibits a slightly divergent flow along an ice ridge with 0.756 m a^−1^ observed surface velocity [1]. The accumulation rate is 64 kgm^−2^ a^−1^ (ice equivalent 7 cm a^−1^) [29]. The annual average surface temperature (10 m-firn temperature) is −45°C and the borehole bottom temperature is −3°C. This leads to bed conditions with subglacial water, which penetrated into the hole during the termination of drilling [24].

Numerical flow models with palaeoclimate forcing have been used to determine an appropriate site for the EDML ice core [30,31] prior to the actual drilling and have also assisted with the dating and interpretation of the palaeoclimate records of the EDML deep ice core [32]. The first two studies [30,31] present temperature and shear strain rates versus depth for their proposed drill sites at 73°57′ S, 03°35′ W and 73°59′ S, 00°00′ E, respectively. As both locations are several hundreds of kilometres away from the EDML site, the given profiles cannot be used for comparison. Although the study by Huybrechts *et al*. [32] is to our knowledge the most recent in-depth flow modelling study for the EDML site, as they apply a higher-order flow model nested in a large-scale model under palaeoclimatic forcing, unfortunately we cannot compare the results, because neither strain rate nor temperature versus depth profiles are reported.

## 2. Methods

### (a) Fabric analysis

**Figure 2.**
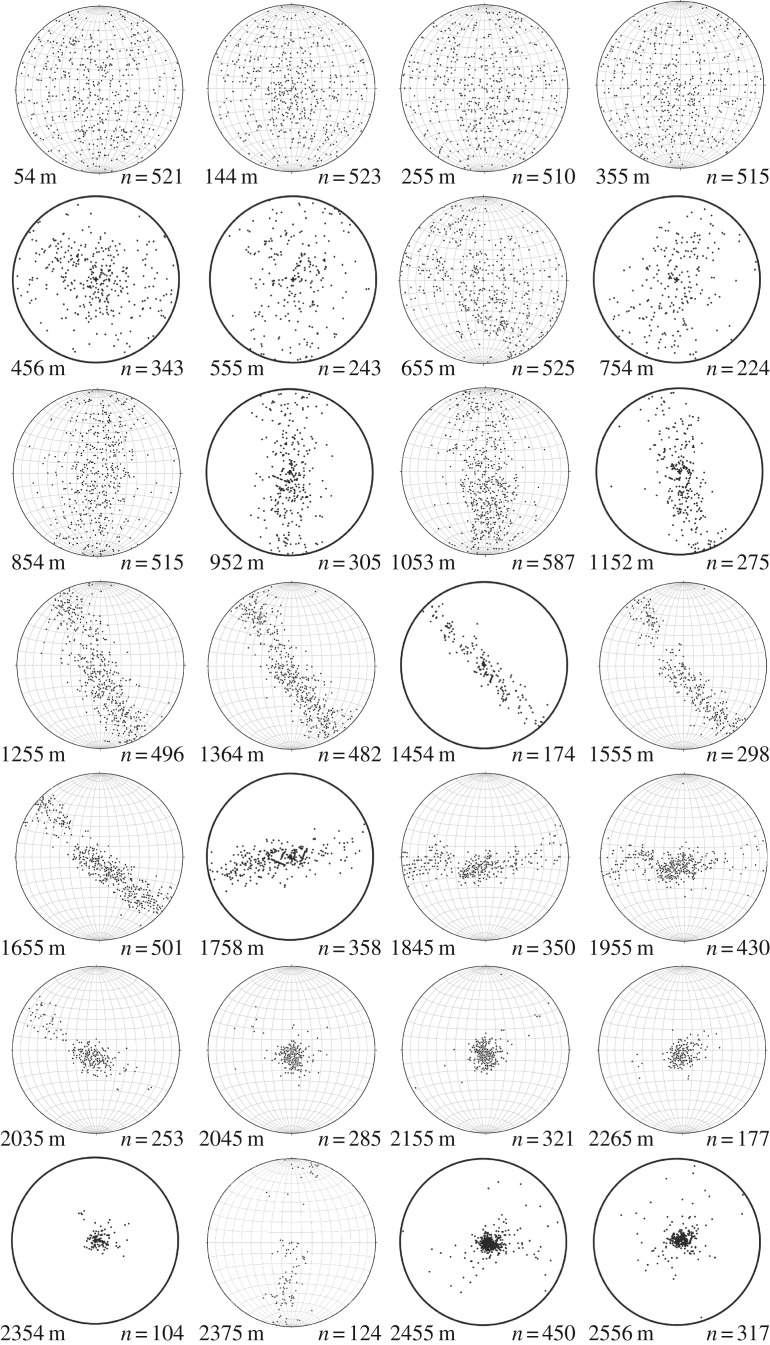
Examples of stereographic projections of the lattice-preferred orientation (LPO). Classical glaciological projection into the geographical horizontal plane (the drill-core axis is at the centre of the circle in each diagram). Pole figures show the classical Schmidt diagram. Gridlines illustrate measurements on vertical thin sections (rotated for this display), whereas diagrams without gridlines originate from horizontal thin sections. Numbers on the left of each diagram give sample depth; on the right of each diagram are the number of *c*-axes (one per grain) plotted. Please note that changes in the orientation of the girdles are related to orientation mismatch between core pieces (drill runs), and not to a sudden change in stress/flow direction.

**Figure 3.**
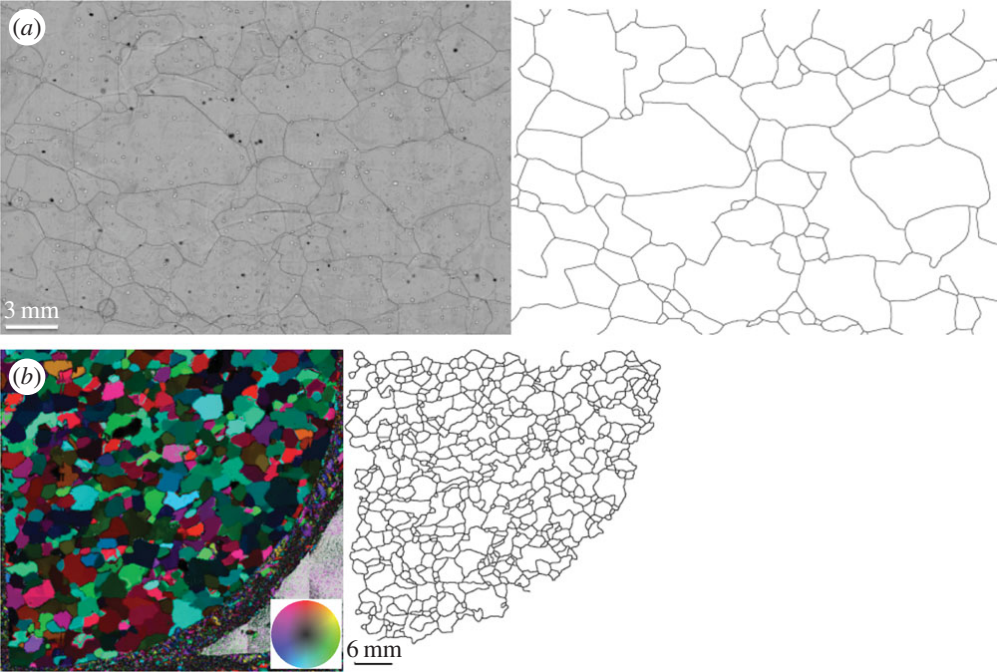
(*a*) Example of a microstructure mapping picture (1553.0 m depth) and the resulting grain boundary network obtained by image segmentation. Left-hand picture shows strong black lines which are grain boundaries revealed by sublimation etching. (*b*) Example of an AVA (Achsensverteilungsanalyse) picture (1056.0 m depth) for LPO measurements (automatic fabric analyser) and the resulting grain boundary network obtained by image segmentation via edge finding. Left-hand picture, false-colour code; see wheel legend.

**Figure 4.**
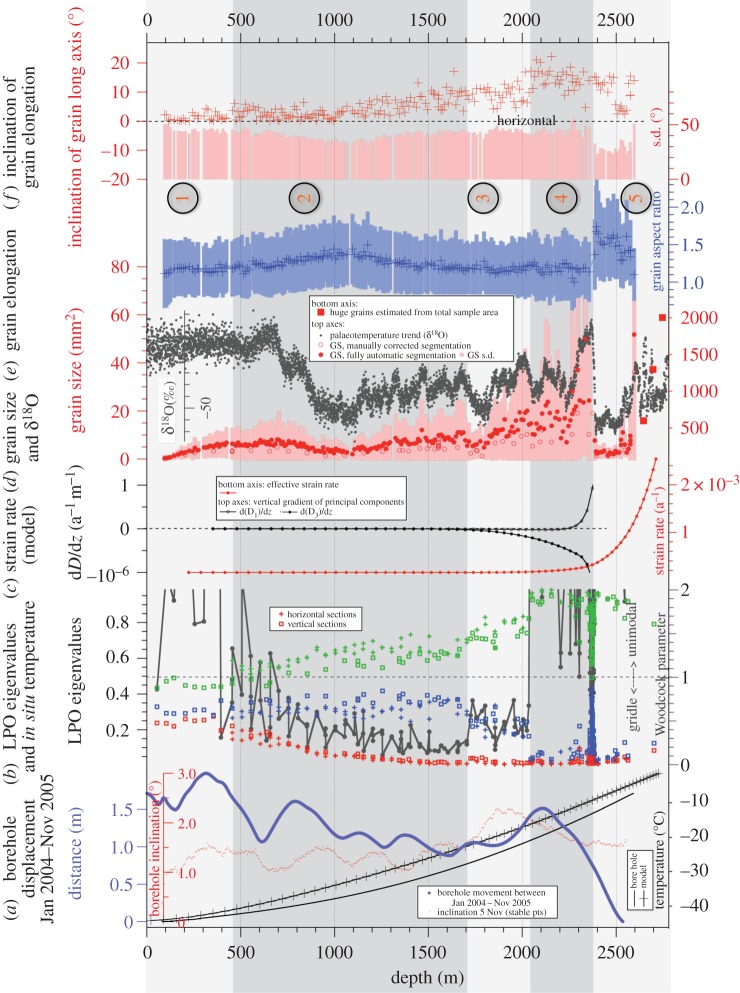
Compilation of ice core micro- and mesostructure data with borehole and modelling data. Shaded regions denote regions of deformation modes explained in the text. (*a*) Borehole data and modelled temperatures. (*b*) LPO data given in eigenvalues of the second-order orientation distribution tensor (red, blue, green) and Woodcock parameter. (*c*) Derivatives of the selected strain rate tensor components derived from the shallow ice approximation model PISM. (*d*) Grain size data from the microstructure map and *δ*^18^O values (‰ standard mean ocean water) from stable water-isotope measurements for palaeotemperature reconstruction (grey). Dataset: doi:10.1594/PANGAEA.754444 down to 2416 m depth. Below 2416 m depth unpublished data (H Meyer and H Oerter 2016, personal communication). (*e*) Grain elongation given as the aspect ratio of the short and long axis of a fitted ellipse. Mean and standard deviation (s.d.) per 10 cm section of microstructure maps. (*f*) Grain elongation directions given as the mean angle of the long axis of the fitted ellipse with the horizontal direction (0°). Corrections, assuming measured dip at 1696 m depth being maximum, lead to lower bound inclinations. Actual three-dimensional inclinations can be higher.

The *C*-axis distribution data (LPO/fabrics; figure 2) are derived from thin sections measured with an automated fabric analyser system of the Australian Russell-Head type [33] that applies polarized light microscopy, where the thin section is placed between systematically varying crossed polarizers [34]. We mainly used the G20 system, measuring approximately 150 vertical and horizontal thin sections in depth intervals of about 50 m (dataset: [35]). For cross- and quality checks, we also applied the improved G50 system for 60 additional vertical thin sections, with partly bag-continuous sampling (dataset: [36]). A few examples of the in total approximately 210 crystal LPO measurements are displayed for illustrative reasons in figure 2 and figure 3*b* as classical pole figures. These so-called Schmidt diagrams show the natural variability among at least 100 measured grains per section. Additionally, as a more continuous display of all thin section data, we calculated eigenvalues of the second-order orientation tensor of the *c*-axis distributions [37,38], which portray the distribution as an enveloping ellipsoid with the eigenvalues being its three principal axes (figure 4*b*, dataset: [39,40]). This method is well suited to quantify a three-dimensional unimodal or girdle distribution of orientation data. The breadth of the distribution is described by the absolute magnitude of the eigenvalues, that is, it describes how many grains are aligned with the preferred direction. Additionally, the Woodcock parameter is given (figure 4*b*; [41]), which lies in the interval [0,1] for girdle LPOs and [1,∞] for unimodal LPOs.

### (b) Grain size and shape characteristics by light-microscopy microstructure mapping in plain and polarized light

In addition to the LPO investigations, further microstructural data such as grain size evolution and shape-preferred orientations (SPOs) gave insight into, and in turn can influence, the deformation conditions and related processes (e.g. [16,42]). The grain size and shape evolution along this core were examined, using approximately 300 vertical and horizontal thick and thin sections (10 m interval). Owing to the preferred sublimation along the grain boundaries, they develop etch grooves on sublimation-polished surfaces that are visible in plain light illumination [43]. These grooves can then be mapped on microphotographs, revealing the grain boundary network. The grain boundary networks were extracted, using fully automatic image analysis, and evaluated for structural parameters of the grains wherever possible ([44], figure 3*a*), with manual corrections when necessary. The data from vertical sections were complemented by grain structural parameters derived from photographs taken between crossed polarizers for horizontal sections (during the fabric analysis procedure). Here, semi-automatic image analysis with edge detection segmented the image into grain boundary networks (figure 3*b*).

**Figure 5.**
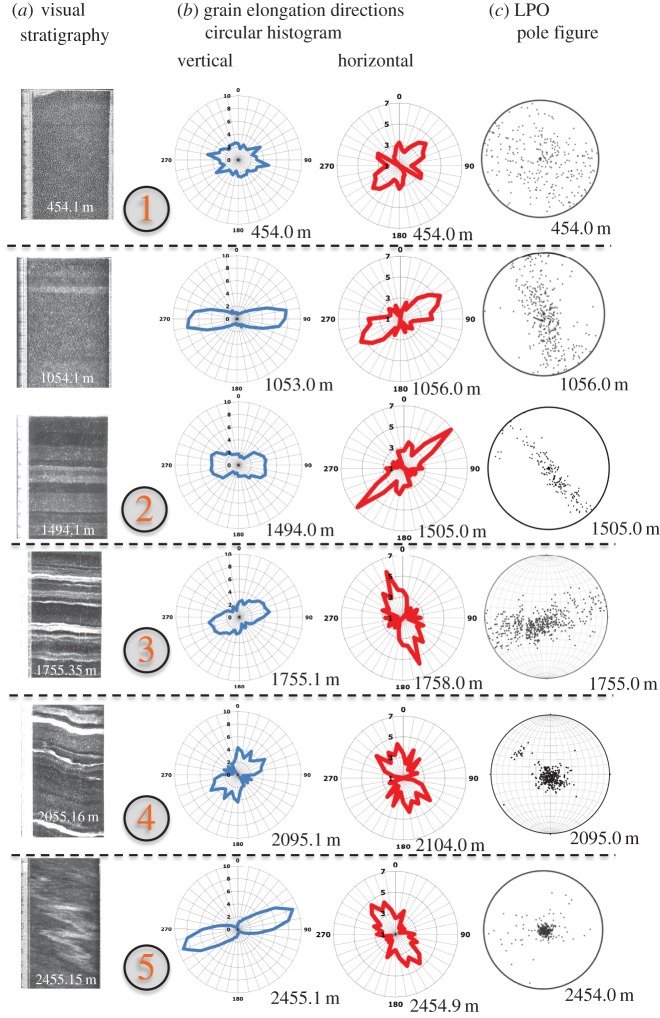
(*a*) Visual stratigraphy line scanner images, selected. (*b*) Distributions of grain elongations given as circular histograms (rose diagrams) measured in sections vertically (right) and horizontally (left) cut from the ice core. Radial axes give the frequency in % with a fixed axis range (7% for horizontal sections, 10% for vertical sections). For vertical sections, directions 0° and 180° are along the core axis. For horizontal sections, azimuthal orientation corresponds to those in LPO pole figures. (*c*) Selected LPO stereographic projections for direct comparison.

From the grain boundary networks, we obtained the two-dimensional grain size, e.g. as areas by pixel counting of individual grains (figure 4*d*) and shape parameters (SPOs). The magnitudes of grain elongations given as aspect ratios (figure 4*e*) and grain elongation directions (figure 4*f*) are calculated from the principal axes of an ellipse fitted to each segmented grain. The distributions of the directions are shown as circular histograms (‘rose diagrams’) in figure 5*b* (display method adopted from [45,46]). Although the drilling process does not allow for an oriented ice core, the relative orientation among individual drill runs has been retained by fitting characteristic core break surfaces during core logging as well as possible and by drawing a continuous ‘top line’ on one side of the core [47]. In the girdle LPO depth range, a change in orientation is identified from the changing direction of the girdle in the stereographic projections (figure 2). However, in the brittle zone (approx. 800–1200 m), where orientation loss happened more frequently, it is less obvious as the data show only a weak girdle pattern in this depth region. The grain elongation direction data reveal possible inclinations from the horizontal and vertical sections (figure 5*b*, §3c). The raw data of inclination measurements from the elongation directions from vertical sections reflect a jump between 1655 and 1758 m (cf. figure 2) owing to a loss in azimuthal orientation of the core during logging, which was caused by a break between 1686 and 1696 m. This jump corresponds to a core rotation of about 40° (cf. figure 2) and significantly masks the signal in the raw measurements of elongation directions. A second recognizable azimuthal loss shows less rotation (10–20°) between 1955 and 2035 m depth, but with higher ambiguity owing to the strongly developed single maximum LPO. The rotational symmetry of the single maximum distribution does not allow a reliable reconstruction of the likely true inclination. However, the effect on grain elongation inclination data is minor. It may however contribute to the increasing variability in this depth range. The 40° jump between 1686 and 1696 m was corrected in the grain elongation inclinations by means of trigonometry, assuming that the steeper inclination at 1696 m is the true dip of the three-dimensional elongation plane. Thus, the data shown here represent a lower bound of the actual inclination. The correction assumes a maximum dip, which is however unknown. Furthermore, only absolute values are given, as the dip direction leading to clockwise or anticlockwise inclination depends on the core and sampling orientation.

### (c) Visual stratigraphy line scanning

Visual stratigraphic layering was recorded continuously along the core with a line scanner (LS) developed at the Alfred Wegener Institute in Bremerhaven, Germany, and the Niels Bohr Institute at the University of Copenhagen, Denmark [48,49].

Typical LS samples are 1 m ice slabs that have been cut lengthwise from the EDML core, with their upper and lower surfaces polished with a hand-held microtome blade. Oblique dark-field, indirect illumination of each slab is scanned along the core axis by a digital line-scan camera. The line-scan camera can capture only the light that has been scattered by inclusions in the ice matrix, such as microscopic particles or air bubbles, because ice itself is transparent. Thus, the degree of brightness recorded by the camera is proportional to the concentration of inclusions in the sample, in such a manner that a clear piece of ice, free of inclusions, appears dark (figure 5*a*).

Images recorded with the LS system reveal the visual stratigraphy of an ice core in great detail (1 pix = 0.1 mm) because of the high resolution and sensitivity of the digital line-scan camera. Strata as thin as 1 mm can be easily detected with this method. Any small change in the optical properties of the ice matrix, usually caused by a change in the mean size or concentration of inclusions, gives rise to a new horizon.

### (d) Borehole logging

During the EDML drilling campaign, the borehole was logged at several stages of drilling progress. The logging system continuously recorded the tilt of the borehole with respect to the vertical (inclination) as well as the heading of the borehole with respect to magnetic north (azimuth) by means of a compass [50]. Additionally, the diameter of the borehole was measured. Here, we use the change of the borehole course (inclination and azimuth; figure 4*a*) owing to the local ice deformation between two measurements (January 2004 and November 2005) to estimate the strain regime at the EDML site. Changes in diameter of the borehole, which do occur, are not evaluated in this study.

### (e) Strain rate estimations from the ice flow model

To derive a velocity–depth profile at the EDML site, we used the parallel ice sheet model (PISM v 0.6.1) [51–53].

The deformation of polycrystalline ice is modelled, using the Nye generalization [54] of the Glen–Steinemann power-law rheology [55,56]. The effective viscosity, which connects the strain rate tensor with the deviatoric part of the Cauchy stress tensor, depends on strain rate, pressure, temperature and water content [57,58], where the last two quantities are diagnostically calculated from the enthalpy field. The enthalpy scheme used in PISM is fully described in Aschwanden *et al*. [59], to which the interested reader is referred.

At each time step of a PISM simulation, the geometry, temperature, water content and basal strength of the ice sheet are included into momentum balance equations to determine the velocity of the flowing ice.

Instead of solving the full set of Stokes equations for the momentum balance, PISM solves, in parallel, two different shallow approximations: (i) the non-sliding shallow ice approximation (SIA [60]), which describes ice as flowing by shear in planes parallel to the geoid, and (ii) the shallow shelf approximation (SSA [61]), which describes a membrane-type flow of floating ice, or of grounded ice which is sliding over a weak base. The ice flow velocity of the grounded ice is computed with a hybrid scheme based on a weighted superposition of both shallow solutions (SIA + SSA), where the SSA solution acts as a sliding law (see [62] for details). The computed three-dimensional velocity field thus contains horizontal longitudinal (membrane) stresses from the SSA solution as well as vertical shear stresses from the SIA solution.

The present-day state of the Antarctic ice sheet was computed based on the present-day geometry Bedmap2 [27], and varying datasets for present-day boundary conditions such as: surface temperature [63–65], surface mass balance [65–67] and geothermal heat flux [68,69] and the update from Purucker [70] based on the method of Fox Maule *et al.* [69]. The original dataset of Fox Maule *et al*. [69] has been capped at a value of 0.07 W m^−2^ according to the SeaRISE-Antarctica recommendations [71]. Using all combinations of the boundary conditions above, with the restriction that RACMO2.3/ANT [65] data for surface skin temperature and accumulation rate are used together for consistency, we have set up an ensemble of 15 different simulations. We have chosen the combination RACMO2.3/ANT surface forcing together with the Shapiro & Ritzwoller [68] heat flux as our reference simulation. Other combinations have been applied to estimate the sensitivity of the model to varying forcing data. We have chosen the same set of parameters within the PISM model that have been used for the ice sheet modelling project SeaRISE Antarctica (see ‘Potsdam’ model in [72]), where the model was forced with constant present-day climate. Although surface temperatures and accumulation rates have changed over time in the palaeoclimate context, we prescribe the constant present-day climate in order to avoid a complete recalibration of the model that would be far beyond the scope of this study. As the temperature field near the base—where most of the deformation takes place—is mainly controlled by the geothermal heat flux, we expect to have a reasonable uncertainty estimate owing to our ensemble set-up.

Each simulation was conducted in a series of subsequent grid refinements (all based on the initial 1 km present-day geometry) using 40, 20 and 10 km horizontal resolution and 41, 81 and 101 vertical layers, respectively. We used the flux correction method provided by PISM in addition to a prescribed (present-day) calving front position to result in an ice sheet that is close to the observed present-day geometry. After initialization (1 a), a short relaxation period (100 a) and a purely thermal spin-up with the geometry held fixed (200 k years) on the 40 km grid using only the non-sliding SIA, the model ran for 100 k years, 20 k years and 4 k years on the 40, 20 and 10 km grid, respectively, in the hybrid (SIA + SSA) mode. The individual boundary conditions that have been chosen for one ensemble member were held constant over time, thus forming a present-day climate equilibrium model realization.

All components of the strain rate tensor as well as the effective strain rate *D*_e_ (figure 4*c*) and the three principal strain rates, D_1_, D_2_ and D_3_ (eigenvalues of the strain rate tensor, figure 4*c*), have been derived from the simulated velocity field diagnostically on the PISM grid.

## 3. Results and data

### (a) Lattice-preferred orientation

The *c*-axis distributions show a typical evolution with depth for a drill site located on an ice divide: a broad distribution represented by all three eigenvalues around one-third (figure 4*b*) appears uniform in the pole figure display (figure 2) and is almost constant in the upper 450 m. Below this, LPO develops continuously into a vertical great-circle girdle distribution by narrowing of the girdle towards three distinct eigenvalues (in the following referred to as e_1_, e_2_, e_3_) down to 1650 m depth. In a transitional region (approx. 1700–2030 m), the LPO evolves towards an elongated single maximum well defined by the inflection point of the e_2_-trend (figure 4*b*): above approximately 1650 m e_2_ grows; below 1650 m e_2_ decreases. Although increasing strictly monotonically with depth in the upper two-thirds of the core, also the e_1_-trend shows a change in slope at approximately 1650 m depth. The gradual evolution of the single maximum is generated by a gradual concentration of *c*-axes within the girdle, observable in panel 5 of figure 2 and a slight re-widening of the central part of the girdle in the pole figure (see also figure 2). This is confirmed by the Woodcock parameter (figure 4*b*), which rises at this depth level towards unimodal distributions. This is followed by a collapse of e_3_ and e_2_ into a single maximum at approximately 2030 m depth (figures 2 and 4*c*). In a narrow layer (from 2345 to 2395 m depth), the LPOs become highly diversified with tendencies of single maximum to girdle and back to single maximum distributions represented by a wide range of values in e_3_ and e_2_ (0–0.5) and in e_1_ (0.5–0.9) (figure 4*b*). This is the lower half of the Eemian (marine isotope stage 5.5 (MIS5.5)) layer, characterized by lower *δ*^18^O values (figure 4*d*), which indicates higher precipitation temperatures than derived from the overlying ice of the last glacial period. Statistical evaluation is difficult, as the Eemian ice exhibits the largest grain sizes observed in the EDML core, except for the basal layer (figure 4*d*).

Systematic offsets of the eigenvalues e_2_ and e_1_ between vertical and horizontal sections (figure 4*b*, e.g. in region 2) are due to the ambiguity of measurement of *c*-axes lying close to the observation plane. Data at the periphery of a pole figure (projection onto a thin section plane) are of low quality and thus partly excluded from the population in display and LPO data processing, leading to the described bias. The automatic fabric analysers used here calculate the extinction angle, where no light gets through the system, by fitting the light amplitude values for each step of the polarizers to a sinusoid curve [73,74]. The low-quality effect of *c*-axes in a plane normal to the observation axes is caused by the G20 instrument often being unable to resolve the quadrant pair of the azimuth of these orientations. The G20 used a rotating prism to realize three viewing axes and images are taken from three directions. This includes a fourfold symmetry of the extinction angles for the viewing directions. The automatic fabric analyser software philosophy excludes any possibly false information, thus any ambiguous data values are not included. The G50 instrument uses a quarter-wave (λ/4) plate to determine the azimuth, so that fast/slow directions by adding or subtracting λ/4 resolve the azimuth symmetry of the quadrants (defined by the crossed polarizers; for further details on crystal orientation and interference effects, see for example [75]). Comparison measurements verified this effect.

### (b) Grain size

The grain size generally increases with depth (0.3–2000 mm^2^), but is strongly affected by the impurity content of ice from different climatic stages (figure 4*d*). Over the first 700 m, the grain size increases and decreases again with depth until reaching the last glacial maximum (LGM) ice (MIS2, approx. 1000 m), which is identified by the most depleted stable water isotope values (figure 4*d* [28,76]; H Meyer and H Oerter 2016, personal communication: stable oxygen isotopes of deep ice core EDML (deeper than 2416 m)). Correlation coefficients for grain size and *δ*^18^O confirm the correlation on this large scale with 0.65 for depths below 700 m and 0.79 for depths below 900 m depth. Below, the mean grain size increases again down to a depth of approximately 1700 m, followed by a steep size decrease down to approximately 1750 m (MIS4). Below this, grains increase again down to approximately 2300 m (Eem, MIS5.5), but show a higher variability within samples than in all stages above. A sharp decrease down to the smallest measured grain size values is observed just below the MIS5.5 ice (approx. 2400 m), followed by a sudden increase (approx. 25 times) to grains of similar or larger size than the section size in the deepest basal ice layers. Further doubling to tripling of grain size occurs in this basal layer estimated from the deepest three samples measured (‘square’ symbol and separate bottom axis in figure 4*d*).

The above-described trend in grain size can be observed fully automatically as well as by manually correcting segmentation of images (excluding the three basal samples). Both methods give diverging results at various levels with the manually corrected segmentation giving approximately 10% smaller values in most depth levels (figure 4*d*). Thus, the fully automatic segmentation may not capture all (slightly weaker) grain boundaries. These weaker boundaries, in principle, are an indication for newly formed grain boundaries developing from subgrain boundaries during dynamic recrystallization ([77]; §4.1 in [78]). This seems to occur along the whole EDML ice core [79]. However, a deeper analysis of these mechanisms, e.g. to decipher the actual processes of grain boundary formation, is only possible in combination with high-resolution full-crystal orientation measurements [80–82], and lies beyond the scope of this work.

### (c) Grain shapes

A visualization of SPO given as grain elongations in the vertical and horizontal sections is shown in figure 5*b,c* as circular histograms (also called ‘rose diagrams’), which show the magnitude and direction of elongation. Statistical evaluation (figure 4*e,f*) is not possible for horizontal sections owing to few samples. In general, only moderate mean aspect ratios up to 1.8 are observed (figure 4*e*). Large standard deviations in all samples indicate that only a few grains are actually elongated, whereas many exhibit an equi-dimensional habitus. However, for vertical sections, we observe clear trends in the mean values: first, increasing elongations from 1 to 1.5 down to 1100 m depth, followed by a decrease down to approximately 1700 m depth. Between 1700 and 2200 m, aspect ratios remain stable at small average elongations of approximately 1.2, with an increased variability observed among samples below approximately 2050 m depth. Low values approach approximately 1 and indicate equi-dimensional grain shapes on average. Most intensely elongated grains in the EDML ice core are observed at a sudden transition of aspect ratio from 1.1 to 1.7, located between 2356 and 2393 m depth. Below approximately 2575 m depth range statistical analysis is not reliable owing to the very large grain size.

Grain elongation directions, defined as angles of the long axis of a fitted ellipse with the horizontal, show large variations within individual sections (figure 8; s.d. in figure 4*f*). Statistical analysis, however, reveals elongation of grains in the horizontal direction (0°) down to approximately 1000 m depth. This is remarkable as between 500 and 1200 m the relative azimuthal core orientation was lost repeatedly in the brittle zone. The geographical azimuthal (N–E, S–W) orientation of the drill core controls the actual observed angle of inclined features. Three-dimensional-sectioning effects of orientations of lines or planes, e.g. during sample preparation as in this study or in geological outcrops, are well known in structural geology [83]. These sectioning effects lead to minimum bound measurements of angles, and isolated single measurements cannot prove horizontal orientation. Repeated sectioning of samples down to approximately 1000 m depth all showing on average 0° angles thus reveal a truly horizontal-preferred elongation direction. On average, grain elongation directions start to incline slightly from the horizontal to a few degrees down to approximately 1700 m. This has to be considered as a lower bound owing to the three-dimensional cutting effect of inclined objects. The inclination of grains progressively increases to about 10° at a depth of approximately 2030 m. Elongation directions appear to stabilize below this.

Comparison of the vertical girdle LPO presented in the standard glaciological stereographic projections into the horizontal (figure 5*c*) with grain elongations in horizontal sections (figure 5*b*, right column) shows that the mean orientation of the girdle plane, that is, the average plane containing all *c*-axes, and the orientation of the grain elongations in the horizontal are perpendicular to each other.

### (d) Visual stratigraphy

The visual stratigraphy in the uppermost 950 m is generally horizontal, straight and faint (figure 5*a*). The last feature results from the fact that air bubbles in this zone are by far the largest and most efficient light scatterers, so that the visual stratigraphy is dominated by depth variations in the number and size of air bubbles. The ice core in this zone appears so bright under the LS that the recorded images seem partially washed out, with a faint stratigraphy. Within the bubble–hydrate transition zone (BHT zone, 800–1200 m [84]), stratigraphic variations in the number and size of air bubbles gradually increase with depth. Below the BHT zone, all bubbles have transformed into air hydrates, which have a refractive index more similar to that of ice and are consequently inefficient light scatterers. Therefore, the ice stratigraphy below 1200 m is defined by variations in the concentration of micro-inclusions (e.g. salt or dust particles). In sufficiently high concentrations, micro-inclusions can scatter the incident light and make the ice seem opaque, forming strata of light-grey appearance of millimetres to decimetres thickness, called cloudy bands [85,86]. Owing to their intensity and frequency, cloudy bands are the main stratigraphic feature of deep (bubble-free) polar ice.

From the lower part of the BHT zone at approximately 950 m depth down to around 1600 m depth, the EDML stratigraphy remains undisturbed and horizontal (figure 5*a*). Just a few cloudy bands occasionally show minimal undulations with amplitudes not larger than a couple of millimetres. Below 1700 m depth, these undulations gradually increase in intensity and frequency, to such an extent that below 1700 m depth microscale folds develop and some flimsy cloudy bands become slightly inclined or disrupted (figure 5*a*). However, it is only beneath 2050 m depth that well-defined mesoscale folds (with amplitudes up to a few centimetres) and sloped strata (inclined up to 15° from the horizontal) become dominant (figure 5*a*). Further down, the intensity of such disturbances increases notably: cloudy bands appear ragged and fuzzy at diverse inclinations, sometimes up to 30° with respect to the horizontal.

Below 2200 m depth, the strong layer mixing and low concentration of micro-inclusions make cloudy bands too faint and disrupted to be identified. Notwithstanding, at 2386 m, cloudy bands suddenly reappear neatly parallel, horizontal and well defined again. Faria *et al*. [15,78,86] have pointed out that this striking stratigraphy change accurately coincides with a sharp increase in impurity content that marks the transition from the last interglacial (MIS5.5) to the penultimate glacial period (MIS6), approximately 130 000 years ago (figure 4*d*). It coincides with conspicuous changes in ice microstructure, e.g. an abrupt reduction in grain size (5 mm to less than 1 mm) and an increase in grain elongation (aspect ratios 1.2–1.7), within a depth interval of less than 10 m (figure 4*d,e*).

Drastic variations in intensity, inclination and folding of cloudy bands are recognized below 2400 m depth. Intense cloudy bands inclined up to 45°, kink folding, multiple *z*-fold and other serious stratigraphy disturbances become frequent. As a general trend, the intensity of the cloudy bands gradually reduces with depth until they completely disappear below 2600 m, where the temperature rises above −7°C and the average grain size increases dramatically (figure 4*d*).

### (e) Borehole data

The measured borehole inclination and azimuth were smoothed, using a Butterworth low-pass filter to eliminate the influence of movements of the cable and erratic movements of the logger owing to unevenness of the borehole walls. The logger depth is taken as the paid out cable length. This set of three coordinates (inclination, azimuth, depth) was then transformed into three-dimensional Cartesian coordinates *x*, *y*, *z* of the borehole track by integrating the data along the path. Owing to the integration, the possible error in the borehole track increases with depth. The data presented in this study were recorded in January 2004 and November 2005. Both logs reached a depth of approximately 2550 m, which is still about 200 m above the bedrock. Later, re-logging data could not be included here owing to a failure of the azimuthal measurement, preventing the calculation of the shape of the borehole. However, these latter data would not have included the lower approximately 200 m, because the intrusion of subglacial water made the lowermost part of the borehole inaccessible. As the water rose faster than it could be removed, this also caused the end of the drilling [24,25].

The calculated borehole displacement and the inclination of the hole are displayed in figure 4*a*. The gradient of the borehole displacement is largest below approximately 2050 m depth (figure 4*a*), indicating the depth where shear becomes dominant. Above, the displacement is fluctuating around approximately 0.5 m, which is partly owing to the rather high noise of these measurements compared with the small displacement signal owing to the slow deformation. The absolute total displacement is in accordance with the measured surface velocity (0.756 m a^−1^ [1]) at the site over 2 years, lacking any evidence of significant basal slip below the EDML drilling site despite the occurrence of subglacial water.

### (f) Strain rate estimates from the model

The simulated temperature–depth profile extracted from the PISM grid at the grid location next to the EDML site (approx. 1.5 km away) is shown in figure 4*a*. Comparison with the measured borehole temperature (same figure 4*a*) reveals a distinct difference, as modelled temperatures are always higher (approx. 5°C, maximum) than the observations. In the lowest part of the borehole, temperature observations are missing, but observed water at the ice–bed interface indicates temperate ice conditions. Thus, the simulated basal temperature of approximately −1.4°C (pressure corrected) underestimates the temperature at the base. The overall curvature of the simulated temperature–depth profile is smaller (more linear) than the observation, most likely indicating that the model underestimates the downward advection of cold ice from the surface. Assuming that the zeroth-ordered stress terms from the momentum balance equations are still the largest contributions to the effective stress at the EDML site, we expect the model to overestimate the deformation of ice, mostly in the ice column except for the basal zone.

The magnitude of the horizontal surface velocity obtained by the model is approximately 1.4 m a^−1^, and is thus twice the observed value (0.756 m a^−1^ [1]), but still within the same order of magnitude. As the base is cold in the model, this surface velocity originates from internal deformation only. The simulated velocity has a stronger northern component than reported in Wesche *et al*. [1]. This corresponds well with the local surface slope from the gridded Bedmap2 dataset ([27], see surface contours in figure 1).

**Figure 6.**
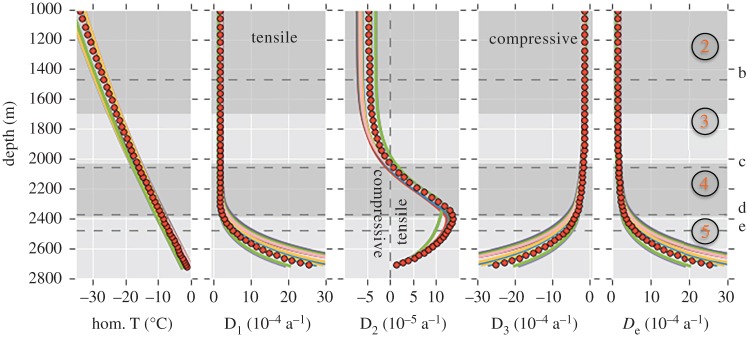
Modelled temperature (pressure corrected), the principal components D_1_, D_2_, D_3_ of the strain rate tensor and the effective strain rate from 15 model runs with varying surface and basal boundary conditions. Our reference simulation is shown as filled red circles, whereas all other simulations are presented as highly overlapping lines with randomly chosen colours. The annotated horizontal dashed lines indicate the selected depth levels for the eigenvectors shown in figure 7. The vertical dashed line in the D_2_ panel is placed at zero strain rate and indicates the transition between compressive and tensile conditions.

Figure 4*c* shows the derived effective strain rate with depth that is approximately constant down to approximately 1700 m depth. The effective strain rate increases between approximately 1700 and approximately 2400 m owing to the increasing influence of shear strain with another significant gain below 2000 m depth, and increases further with a higher gradient below approximately 2400 m. The derived first principal strain rate D_1_ (with D_1_ > D_2_ > D_3_) is always positive (tensile) along the depth profile and increases towards the base up to approximately 420 × 10^−5^ a^−1^ (figure 6). The third principal strain rate D_3_ is always negative (compressive) along the depth profile with the minimum of approximately −412 × 10^−5^ a^−1^ at the base. This component mostly compensates D_1_ (D_1_ + D_2_ + D_3_ = 0, incompressibility condition for ice). The second principal strain rate D_2_ is one magnitude smaller than D_1_ or D_3_ and reaches the maximum (14 × 10^−5^ a^−1^) at approximately 2400 m depth. Although relatively small, the differences between D_1_ and D_3_ towards the bed result in the slightly positive (tensile) component D_2_. D_2_ increases at approximately 1700 m depth evolving from compressional towards tensile and crosses the compressional/tensile border at approximately 2020 m depth, further intensifying its tensile nature. With depth, D_2_, decreases towards zero again. Additionally, the vertical gradients of the strongest principal strain rates D_1_ and D_3_ are shown in figure 4*c* to highlight the onset of the evolution of changing deformation at approximately 1700 m depth.

**Figure 7.**
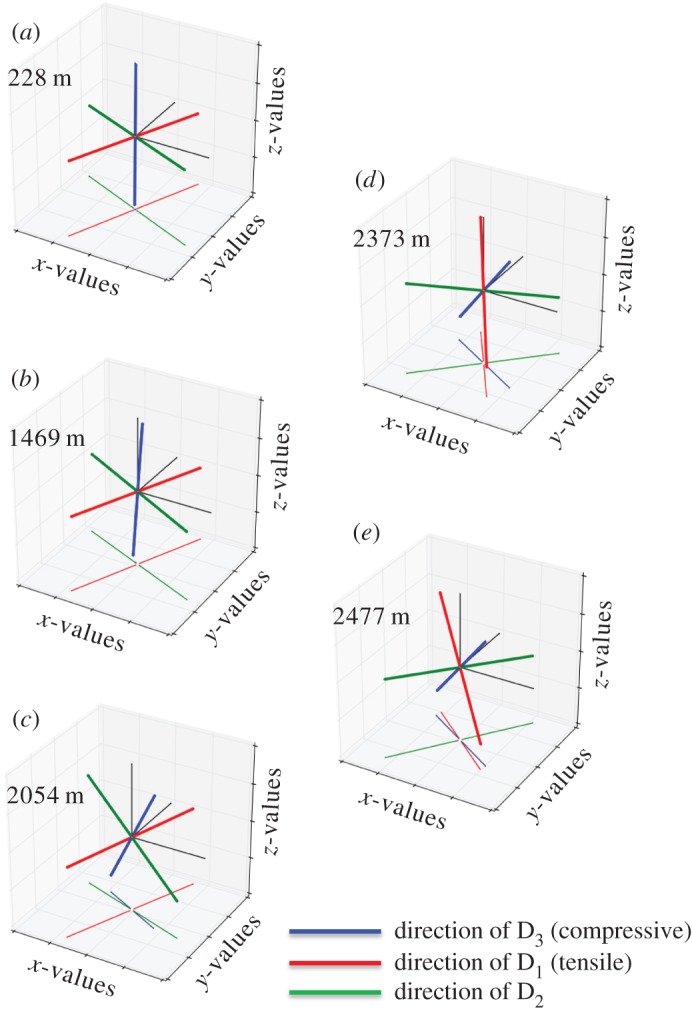
(*a*–*e*) Normalized strain rate eigenvectors at selected depth levels. Directions with respect to the polar stereographic projection (EPSG: 3031). The vectors are projected also to the basal plane of the coordinate system to avoid visual misinterpretation. *x*, east; *y*, north; *z*, up.

The three-dimensional orientations of strain rate eigenvectors are presented in figure 7. In shallow depths, the orientation of D_3_ (compressive, blue) is vertical, D_1_ (tensile, red) is orientated perpendicular to the ridge, and D_2_ lies parallel to the ridge (figure 7*a*). Down to approximately 1000 m depth, the overall deformation can thus be described as a classical extensional regime as expected on ice divides: extension normal to the ridge, almost no deformation, that is, very small D_2_ (figure 6) along the ridge and vertical compression (triaxial deformation). At approximately 1000 m depth, the direction of D_3_ (compressive, blue) and D_2_ begin to tilt by rotation around the D_1_ direction perpendicular to the ridge (figure 7*b*). At approximately 1700 m depth, D_2_ starts to increase (figure 6, central panel), whereas its orientation continues to rotate within the ridge plane around direction of D_1_. This D_2_ increase describes a development from compressive towards tensile nature of this eigenvector. With this, the overall deformation mode changes from a classical divide (extensional) regime towards the basal regime (simple shear). This is also visible in the increasing obliqueness of D_3_ away from the vertical (figure 7*b* versus *c*) and a further decreasing value of D_2_ (figure 6). At approximately 2030 m depth, the D_2_ value reaches zero per year (figure 6, central panel), which can be described as a confinement in this direction and thus indicates almost ideal simple shear in the non-coaxial plane strain. However, this situation only holds for a very thin depth layer, as it is caused by a transition back to triaxial deformation (general shear) with increasing D_2_ towards positive, thus tensile, values (figure 6). D_3_ (compressive) is now approximately 45° inclined (figure 7*c*). At approximately 2360 m depth, D_1_ (tensile) finally rotates away from the direction perpendicular to the ridge. D1 (tensile) rotates quickly between approximately 2370 and 2380 m depth (figure 7*d*) to a NW–SE orientation. Below this D_1_ (tensile) and D_3_ (compressive) the axes are both approximately 45° with the basal bedrock plane (figure 7*e*) and D_2_ is now tensile, though still very small. This describes the situation as expected for bed parallel shear.

## 4. Discussion

The described microstructural parameters from LPO measurements, grain size and elongation distributions as well as the mesostructural characteristics from layering in the visual stratigraphy record can be interrelated in five depth regions along the EDML core. We combine these results with borehole logging observations and strain rate evolution with depth provided by numerical ice sheet modelling (PISM) from the surrounding macroscopic geometries and balances. The combined evaluation of these data shows that the structural observations can be interpreted as the effects of the transition from vertical compression with transverse extension to horizontal shear. The depth horizons are indicated as grey regions in figure 4. It is worth noting that the boundaries of these regions should not be understood as sharp borders in all cases. Thus, depth indications do slightly deviate, because with this multi-parameter approach slightly different reaction times of the deformation and recrystallization processes forming the observed responses may lead to slightly deviating characteristic depths.

By the chosen model spin-up, we ensure that the ice sheet geometry corresponds to the current state. This is essential for realistic strain rates at the borehole location, as the main drivers for ice flow are local ice thickness and surface slope. However, our model set-up results in a temperature versus depth profile that deviates from the observation. Nevertheless, to the best of our knowledge, no Antarctic-wide modelling study and free evolving geometry with palaeoclimate forcing has shown to result in realistic present-day geometry as well as realistic temperature–depth profiles as observed at ice core locations. This is the subject of ongoing research (e.g. ice sheet model intercomparison project (ISMIP6) [87]).

The first panel of our central figure (figure 4*a*) shows the temperature derived from the modelling results in comparison with the temperatures measured in the borehole. The reasons for the difference between modelled and measured temperatures are threefold. First, the present-day climate forcing does not include variations in accumulation rates or surface temperatures over time. Second, the relatively smooth bedrock topography on the 10 km model grid may hinder the model to generate realistic horizontal flux divergence that influences the amount of downward advection at the EDML location. Last, without the flux correction method applied to the surface mass balance the resulting ice thickness tends to be too large at the EDML site (approx. 200 m, figure 2d in [72]). The correction for this (a reduced surface mass balance) causes a reduction in vertical advection which affects the shape of the temperature profile.

Although the simulated temperatures deviate from the observations, they better represent the reality than the parametrized temperature depth profile used in Seddik *et al*. [11], where the temperature is assumed to be constant (approx. −43°C) down to two-thirds of the ice column and to increase linearly below that down to bed (pressure melting point). Furthermore, Seddik *et al*. [11] prescribe the depth of the onset of significant shear deformation by (i) prescribing a Dansgard–Johnsen-type profile for longitudinal strain rates and (ii) the prescribed temperature profile that allows relevant shear deformation only below two-thirds of the column. Bargmann *et al*. [12] followed a similar approach, but implemented the measured borehole temperatures into their one-dimensional model. However, the main aim of Bargman *et al*. [12] and Seddik *et al*. [11] was to model the evolution of the ice LPO, whereas we use an isotropic model to confirm our interpretation of the observational data by considering the full three-dimensional flow field influenced by the surrounding bedrock and surface topography.

### (a) Region 1 (approx. uppermost 450 m)

In the upper part of the ice core, the still small strain rates do not suffice to align the *c*-axes, which are observed to have an almost random distribution, and to produce SPO of grains. This missing SPO with the long axis of grain elongation directions pointing to various directions in shallow depths has been similarly reported for the Dome Fuji and Dome C ice cores [45,46,88]. Deformation of the air–ice composite material in this depth range is facilitated by the compression of bubbles. This agrees well with the expected depth for the dissociation pressure of air–clathrate hydrate at EDML and the observation of the first clathrates [89]. The compression of bubbles leads to a linear relation of bubble size with depth [90]. At first inspection, most points of the ice matrix seem to show only subtle traces of deformation (figure 4*b,d,e,f*), but higher-resolution analyses reveal that highly localized recovery and recrystallization occur already in these shallow depths [77,81,91]. This indicates that deformation is inhomogeneous on the microscale, especially close to air bubbles (§3c and appendix B in [78]), as predicted also by microstructural modelling [92]. Flow model calculations yield low macroscopic strain rates (figure 4*c*), which is in accordance with the average small change with depth of borehole displacement down to 2000 m depth. The rather large wiggling of the borehole data in this upper depth range could in principle be attributed to changing rheology or temperature; however, the borehole displacement data are rather noisy as expected owing to the small surface velocity. With repeated borehole logging after 10 years, we expect more robust data with a better noise–signal relation.

Under these conditions, grain boundary migration dominates the microstructure evolution and masks the deformation habitus of grains [18,19]. This has been observed with respect to the appearance of triple junctions [17] as well as grain boundary irregularities [77]. Identification of deformation kinematics by the available data is therefore not possible in this depth region. A statistical analysis of bubble shape or distribution would be possible but challenging owing to the high variability. First experiences, e.g. with micro-computer tomography measurements, show that the statistical problem is significantly larger with bubble observations [93] than with the microstructural observation shown in this study. It can be speculated that compression owing to overburden pressure of newly accumulating snow prevails, but the observed bubbles are of round shape in the vertical and horizontal sections. The reason for the bubble behaviour is that evaporation–precipitation equilibrium processes inside air bubbles act much faster [94] than the slow strain rate deformation and thus cannot notably change the bubble shape.

In this depth range, the overall deformation yields a classical extension regime as expected on ice divides: extension normal to the ridge, almost no deformation along the ridge and vertical compression (triaxial deformation; figures 6 and 7*a*).

### (b) Region 2 (approx. 450–1700 m depth)

The progressive evolution of a vertical girdle LPO (figure 4*b*) and simultaneous strengthening of grain elongation perpendicular to the LPO girdle (figure 5*b,c*) and along the horizontal plane suggest the dominance of a horizontal extension as described before by Lipenkov *et al*. [95]. Grain elongation directions are parallel to the horizontal, although the azimuthal orientation of the core was repeatedly lost in the brittle zone, leading to a random sample cutting direction. This indicates true horizontal elongation down to approximately 1000 m depth and thus triaxial deformation with one dominating extensional component.

Flow model calculations predict that at approximately 1000 m depth the compressive direction (D_3_) starts to incline away from the vertical (figure 7*b*), which is indeed reflected in the SPO as the dip of grain elongation becomes inclined from the horizontal to several degrees (figure 4*f*), whereas the borehole was inclined by only approximately 1.5° throughout this region and below down to approximately 1700 m depth (see borehole inclination in red in figure 4*a*). The microstructure develops aspects of ‘oblique foliation’, typical for shear zones in quartzite rocks [16]. This is caused by the principal deformation axes leaving the geographical vertical–horizontal orientation towards an inclined one, and can be interpreted as a first effect of a small shear component becoming relevant. The effects of this are still subtle, and only visible in the grain shape, without any influence on the visual stratigraphy, which remains intact (figure 5*a*).

In this region the grain size also decreases with the changing type of ice (Holocene to glacial), which has also been described for most other deep ice cores [85,95,96].

### (c) Region 3 (approx. 1700–2030 m depth)

**Figure 8.**
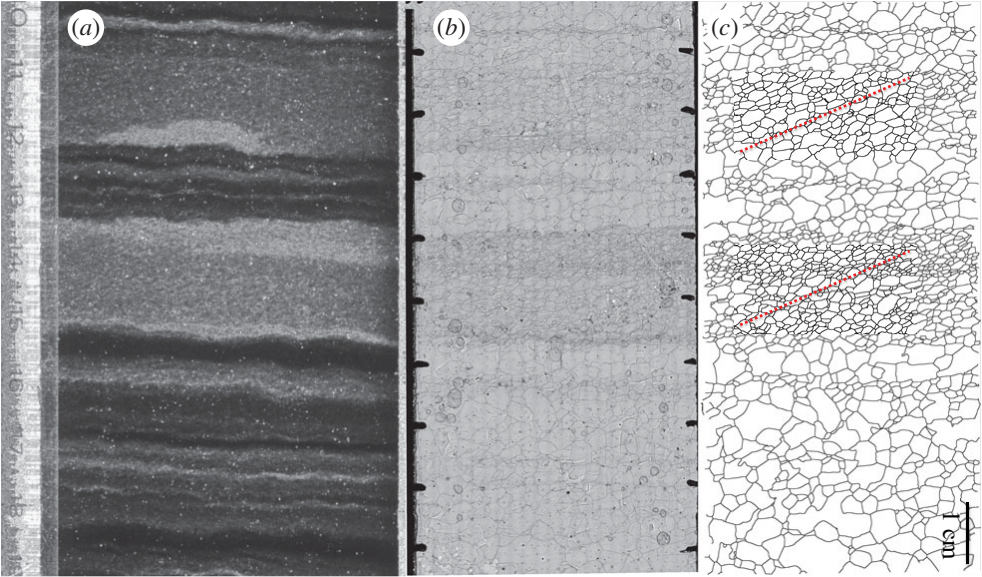
Example ‘oblique foliation’ characterized by inclined, elongated grains in ice (1785.0–1785.1 m depth). (*a*) Visual stratigraphy section. (*b*) Corresponding sublimation etched surface microstructure map. (*c*) Resulting segmented grain boundary network with the approximate inclination of maximum obliqueness sketched (dotted red line) in cloudy bands. Note that the measurement data of this obliqueness given in figure 4*f* average over the whole 10 cm section, which results in a smaller value. (Online version in colour.)

The successive evolution from the vertical girdle LPO towards a vertical single maximum LPO (figures 2 and 4*c*) indicates a transition from dominating triaxial deformation towards horizontal shear. Flow model calculations show that D_2_ starts to increase (figure 6) at approximately 1700 m while rotating within the ridge plane. D_2_ development from compressive towards tensile (figure 6) and increasing obliqueness of D_3_ (figure 7*b,c*) describe the change from the extensional regime typical at ice divides towards an increasing shear contribution. The transition from dominating triaxial deformation towards horizontal shear causes an increase in the dipping angle of grain elongations in vertical sections up to 10°, forming a microstructure close to the ‘oblique foliation’ observed in figure 8. This angle is well above the observed borehole inclinations, which also at this depth are less than 2° shortly after coring. This microstructure notably appears more pronounced in so-called cloudy bands with a higher impurity load. This can be interpreted as an implication for the higher influence of deformation versus recrystallization on the microstructure, owing to either strain localization or inhibited grain growth [97]. The further destabilization of the dominance of triaxial deformation produces millimetre-scale undulations in the visual stratigraphy, because the principal compression direction becomes inclined towards 45° during bed-parallel shear deformation. The successive transition depths from triaxial deformation with horizontal extension and vertical compression to shear deformation suggested by these structural observations are in accordance with predictions by the flow model. Model predictions show a decreasing compressive component (D_3_) at approximately 1700 m depth (cf. vertical derivatives in figure 4*c*), whereas the tensile component D_1_ remains approximately constant. This deviating evolution of D_1_ and D_3_ is clearly represented by the increasing D_2_, which develops from slightly compressional towards extensional behaviour in this depth region (figure 6). The low strain rate is also reflected by the upper part of the borehole (down to approx. 2030 m), as observed in January 2004 being hardly tilted until November 2005.

At the transition from region 3 to region 4 at approximately 2030 m depth, model predictions suggest plane strain deformation as almost ideal simple shear with D_2_ being zero per year (figure 6).

### (d) Region 4 (approx. 2030–2385 m depth)

The single maximum LPO along the vertical core axis is fully developed after a sudden final collapse of the girdle between 2035 and 2045 m. This sudden change in the LPO was also detected as a clear reflector in radio-echo sounding (RES) data, which is caused by the dependence of the electromagnetic wave velocity from the crystallographic orientation [98–100]. Grain elongation direction histograms derived from vertical sections (figure 5*b* left) show a broad, but distinct distribution (cone angle of 45°) with a slight tendency towards double/multiple maxima. This can be interpreted as grains being partly elongated perpendicular to the main compression direction in shear, thus lining up in the instantaneous stretching direction [16], and grains partly being further rotated towards the shear plane [18,19]. These multiple maxima in the SPO description should not be confused with multiple maxima in the LPO. Elongation of grains is caused by deformation. Nucleation usually serves as an explanation for the multiple maxima of LPO, but does not induce grain elongation. In this region, the overall deformation changes again to triaxial deformation (general shear) with increasing D_2_ (figure 6). The principal compressive direction (D_3_) is now approximately 45° inclined. The increasing component of bed-parallel shear readily causes millimetre-scale *z*-folding by amplifying small undulations, which leads to local tilting of stratigraphic layers (10–15°). RES reflectors fade out in this depth (‘echo-free zone’) owing to the loss of layer coherency [101] caused by the intensely disturbing flow characteristic for bed-parallel shear deformation [102].

The bottom layer of region 4 (approx. 2365–2385 m depth) is characterized by the lower border of the Eemian ice (MIS5.5) layer at approximately 2360–2390 m depth (figure 4*d*). However, being approximately 400 m above bedrock, this layer is nearly at the same altitude as the bedrock heights just downstream of EDML, as depicted in figure 1: the EDML site lies on a region at approximately 100 m a.s.l. bedrock altitude, whereas the bedrock approximately 50 km downstream elevates to approximately 500 m a.s.l., so that the ice in this depth flows against it. This may have an effect upstream as the ice has to flow around this. The abrupt jump to smaller grain sizes (figure 4*d*) at this transition is classically explained by impurity influence on grain size [39,103–106], which is confirmed by the correlation coefficients of grain size with isotope measurements [107]. Just above the depth of the sudden changes in grain size and shape, we observe a high variability in the fabric data over short depth intervals. In a layer of less than 20 m thickness around 2375 m depth, multiple maxima LPO and girdle LPO occur with strong variations in neighbouring samples. As the ice is of interglacial origin with low impurity content at this depth, we observe large grain sizes, which in turn raises questions about the statistical significance of LPO measurements and LPO eigenvalue calculations. However, the variations in different types of ice (*δ*^18^O taken as the ‘proxy’ for ice types) in some layers seem to allow grain size to increase locally. The difference in these ice types is characterized by varying impurity concentrations. We use a ‘proxy’ for the ice types however, because picking one impurity is not meaningful with our current knowledge. This may be due to the combined effects of several impurities, as suggested by Fitzpatrick *et al*. [108]. Impurities and their effect on grain size is often explained by the ‘slowdown’ or ineffectiveness of grain boundary migration by changing the grain boundary mobility [104,106]. This phenomenon acts on the microstructural scale (grain and subgrain scale), whereas good correlations are well known on larger scales only (e.g. our grain size–ice-type ‘proxy’ correlation with *δ*^18^O). Direct microstructural evidence on the microstructural scale, such as impurity accumulation along grain boundaries, is difficult to prove [109,110]. This effectively changes the deformation–recrystallization balance under increasing strain rate [18,19,79]. The step in grain elongation magnitude (figure 4*e*) suggests an increased influence of deformation on grain topology moving the microstructure from a recrystallization dominated one towards a deformation microstructure (see a comparison of end members in figure 2 in [19]). Furthermore, although the ice is mainly clear, we see some isolated cloudy bands that are strongly folded, which is an effect of the very strong shear deformation. The characteristic deformation microstructure and strong folding of cloudy bands suggest an alternative hypothesis: localized strain in certain layers. This is in accordance with the macroscopic deformation setting, which predicts an increased strain rate at that depth by the model (figure 4*c*) as well as a qualitative observation of a closing borehole [24]. To assess this hypothesis, further detailed studies on the processes of ice interacting with impurities are needed.

The boundary zone between regions 4 and 5 at approximately 2380 m depth is to some extent a remarkable layer with respect to the overall deformation predicted by the model: the principal tensile direction (D_1_) has left the direction perpendicular to the ridge and rotates very quickly to an along-ridge orientation (figure 7*d*).

### (e) Region 5 (greater than approx. 2385 m depth)

The top of region 5 (approx. 2385–2405 m depth) is characterized by its stratigraphy, which is well defined, ordered and seemingly straight. This is slightly surprising in contrast to the significantly disturbed layers just above. However, we also observe small but sudden changes in the layer inclination and flattened *z*-fold that may suggest that folding has occurred in the past or on other scales which are difficult to evaluate from ice core information. Possible changes of the predominant deformation mechanism showing microshear deformation have been interpreted from striking microstructure observations (slanted brick wall pattern [86,111]). This is in accordance with the extremely high grain shape values in figures 4*e,f* and 5*b*. In spite of the high *in situ* temperature (figure 4*a*) around −10°C, the grain size in the depth range 2385–2450 m does not increase, possibly owing to the high impurity content and shear rate, but increases to very large sizes below 2500 m depth.

Only below approximately 2405 m depth, we observe strong kink folding of the stratigraphic layers, which is a clear indication for high shear rates in recent times. The single maximum LPO slightly inclined from the vertical (figure 2) observed in this narrow transition zone and extreme grain elongations up to aspect ratios of 1.8 (figure 4*e*) are also indicative for high shear rates. Shear shows stronger effects on microstructure as under higher strain rates the balance between deformation-induced and recrystallization-induced effects is changing significantly in favour of deformation (figure 4*c*).

The bottom of region 4 as well as the top of region 5 represent a very interesting, but complex, boundary zone. We want to emphasize the ambiguous depth of this boundary, in the sense that different parameters differ strongly in the zone between region 4 and region 5: (i) the LPO changes and shows anomalous behaviour between 2365 and 2380 m depth, (ii) grain shapes elongate suddenly between 2385 and 2395 m depth, (iii) grain sizes change at the lower Eemian layer border (2356–2386 m depth) and show (iv) grain growth only from 2520 m depth again, and stratigraphy shows anomalous straight banding between 2385 and 2405 m depth. The processes and predominating deformation regimes in this region are not yet understood as they are strongly punctuated and probably catalysed by strong changes in material properties owing to different impurity contents in glacial–interglacial–glacial ice layering. Further investigations on impurity effects on rheology and microstructure are needed.

In the flow model, in region 5, tensile and compressive axes are both at approximately 45° with respect to the bedrock, with a small tensile component D_2_ (figure 6), as expected for bed-parallel simple shear.

At this point, it has to be emphasized again that the slight depth deviations of the region borders in different parameters are probably owing to slightly different reaction times and feedback loops of the processes forming the responses by changing the material in the micro- and mesostructure.

## 5. Conclusion

Detailed observational data from the ice core micro- and mesostructure can be interpreted to constrain the main deformation modes (e.g. compression versus shear) along the ice column. We use an isotropic model to confirm this interpretation by considering the overall three-dimensional flow field driven by ice sheet geometry. By combining several parameters from SPO and LPO, we find indications for the deformation and recrystallization processes being active at the EDML site. We show that it is the balance of both which determines microstructure and possibly flow behaviour.

Recent microstructural modelling studies [18,19,112] combined deformation by a viscoplastic full-field approach (taking strong crystal anisotropy into account [113]) with recrystallization (dynamic and static, continuous and discontinuous [114]). These simulations suggested that the effect of recrystallization on the LPO should be minor. The model set-ups have been chosen for comparison with clean and cold polar ice, where, for example, nucleation is supposedly rare. Notwithstanding, under certain conditions, such as high debris load or high temperatures, effects of discontinuous recrystallization can occur [115]. Observations of recrystallization effects on LPOs in ice cores, however, often suffer from poor statistics, because they are limited to the lowest layers, typically characterized by very large grains ([15] and references therein). As mainly unimodal or girdle LPO occurs at EDML, i.e. only a narrow layer (bottom layer of region 4) with multi-maxima LPOs has been observed, we suggest that the LPO at EDML is affected mainly by deformation, and thus the transition between the regions described above appears most clearly in the LPO fabric data. In contrast, grain shape data show rather subtle deformation trends, as they are strongly overprinted by recrystallization. These subtle trends are, however, consistent with the interpreted deformation modes from LPO.

Impurities can have an impact on the balance between deformation- and recrystallization-induced changes in grain topology. Impurities are postulated to affect recrystallization, by slowing down grain boundary migration through pinning or dragging, and deformation itself by providing dislocation sources. Polar ice is always at a high homologous temperature (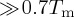), which leads to high recrystallization activity through rotation recrystallization and strain-induced grain boundary migration recrystallization.

Evaluation of the LPO, grain elongation distributions and visual stratigraphy leads to a division into five distinct regions along the core. Here the results are interpreted as the effects of triaxial deformation with horizontal extension changing towards bedrock-parallel shear. This is in good agreement with modelled strain rate trends as well as borehole deformation observations.

Down to approximately 1000 m depth, triaxial deformation with vertical compression and horizontal extension clearly dominates. The influence of shear on the grain structure and fabric starts at approximately 1000 m depth and becomes more prominent between 1700 and 2030 m depth, intriguingly observable in the smooth transition between girdle and single maximum LPO and in the borehole geometry. A final collapse of the eigenvalues in a narrow zone between approximately 2030 and 2050 m depth marks the transition to bed-parallel shear. Shear is the dominating deformation down to the base, but is interrupted by a narrow layer with changing conditions, with abnormal LPO leading into a region of high shear activity, with extreme values in grain shapes.

Owing to relatively small strain rates at the drilling location on the ice divide, only subtle changes in SPO can be observed at EDML, but we suggest including these analyses in future ice core studies especially in mechanically more active regions such as the forthcoming East Greenland ice core project. These analyses can help to assess the share of ice deformation and basal sliding with respect to transport of ice towards the ocean.
